# Comparison between two post-dentin bond strength measurement methods

**DOI:** 10.1038/s41598-018-20891-3

**Published:** 2018-02-05

**Authors:** Laikuan Zhu, Yuping Li, Yung-Chung Chen, Carola A. Carrera, Chong Wu, Alex Fok

**Affiliations:** 1 Department of Endodontics and Operative Dentistry, Ninth People’s Hospital, Shanghai Jiao Tong University School of Medicine, Shanghai, 200011 China; 2 Shanghai Key Laboratory of Stomatology & Shanghai Research Institute of Stomatology, National Clinical Research Center of Stomatology, Shanghai, 200011 China; 3 Minnesota Dental Research Center for Biomaterials and Biomechanics, School of Dentistry, University of Minnesota, Minneapolis, MN 55455 USA; 4 Institute of Oral Medicine, College of Medicine National Cheng Kung University Taiwan, Tainan, 701 Taiwan; 5 Division of Biostatistics, School of Public Health, University of Minnesota, Minneapolis, MN 55455 USA

## Abstract

The push-out (PO) test and the diametral compression (DC) test were performed to compare the merits of two post-dentin bond strength measurement methods. Compared with the push-out test, the disk in DC provided post-dentin bond strength measurements that were more precise. The load-displacement curves from the DC test were much smoother and more linear up to the point of fracture when compared to those from the PO test. Compared to the PO test, DC is easier to perform for determining the bond strength between posts and dentin. No specimen alignment is needed in the DC test, and it produces a smaller standard deviation in the measured bond strength. The main disadvantage of the DC test, however, is that finite element analysis (FEA) is required to calculate the bond strength. The shear bond strength given by the PO test based on the simple formula is not valid, though, and the peak failure load is dependent on friction at the post-dentin interface.

## Introduction

Teeth with extensive loss of coronal hard tissue after endodontic treatment require the placement of posts inside the root canals to support the restorations above^1^. Post loosening and root fracture are the most common reasons for the failure of such endodontically treated teeth^2^. With the post debonded from the surrounding dentin, very high stresses can be created by the occlusal forces in the restored tooth, leading to root fracture^3^. Therefore, a strong and durable bond between the intracanal post and root dentin is vital for the longevity of the restored tooth.

The bond strength between intracanal posts and root dentin can be determined by several mechanical tests; for example, the microtensile test^4^, pull-out test^5^ and push-out test^6^. The specimens used for the microtensile test have to be trimmed to a complex shape, which makes them prone to pre-test failures^7^. In contrast, the specimens used for the push-out (PO) test require minimal machining, and they appear to have a more homogeneous stress distribution around the post circumference when examined using finite element analysis (FEA)^8^. Therefore, the PO test is commonly used for evaluating the bond strength of intracanal filling materials^8–10^. However, it has some limitations. First of all, FEA has shown that the fracture-initiating stress is tensile rather than shear, thus invalidating the formula used to calculate the bond strength. Second, the measurements it produces have a large standard deviation^11,12^. Third, PO bond strengths could be affected by geometric factors of the specimen and loading jig^13^. Further, friction between the post and dentin causes the push-out force to continue to increase after the initial fracture^14,15^. Therefore, the bond strength measured by the PO test, based usually on the maximum load, is likely to be an overestimate.

The diametral compression (DC) test, or the Brazilian disk test, has been widely used for measuring the tensile strength of brittle materials. The stress distribution and failure mode in the specimen have also been investigated in detail^16^. A modified DC test has successfully been used to measure the dentin bond strength of intracanal posts and direct composite restorations^17,18^. The bond strengths determined by this test seemed to have lower coefficients of variation (~15%).

The purpose of this study was to compare the merits of the DC and PO tests and to try to offer an appropriate method to measure the bond strength of intracanal post with root dentin. The null hypothesis was that there was no significant difference between the two methods to measure the post-dentin interfacial bond strength.

## Results

### Load-displacement curves

**Figure 1:**
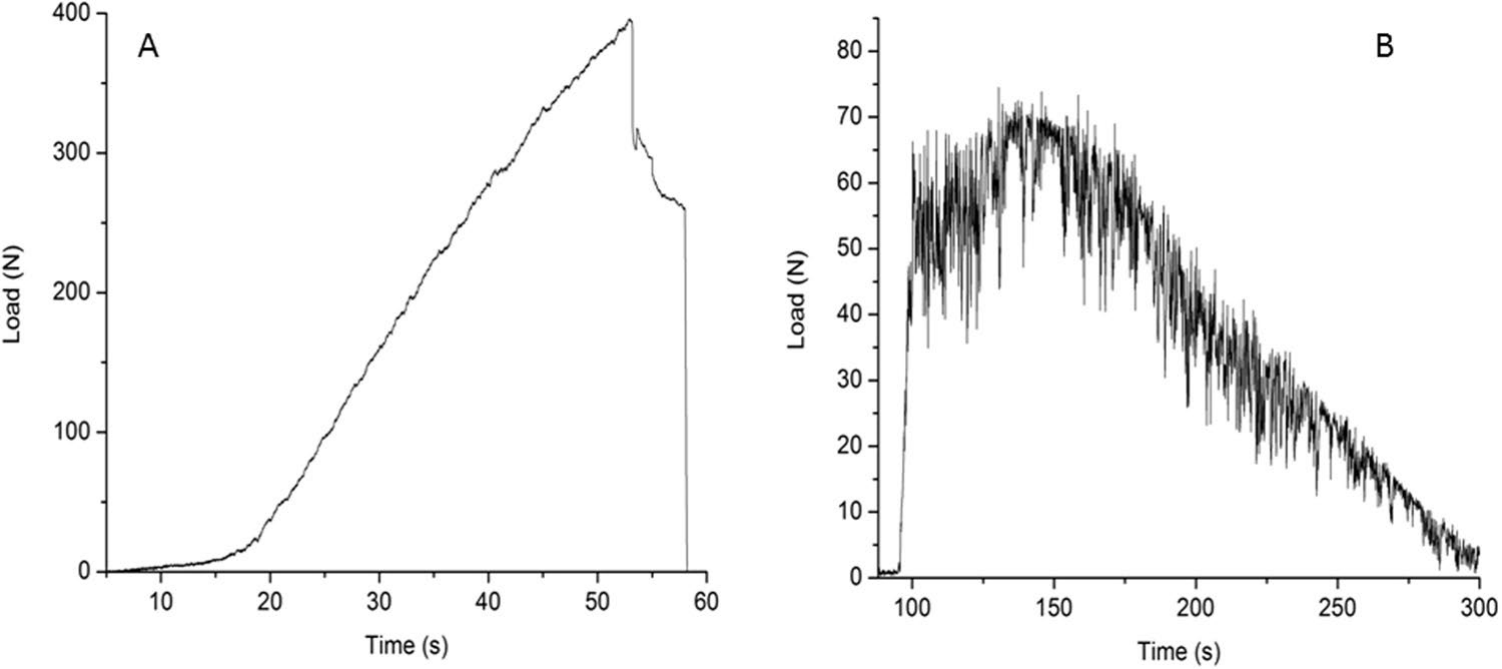
Typical load-time curves from the mechanical tests. (**A**) from a DC test; (**B**) from a PO test. Note that displacement = time × 0.5 mm/min.

Load-displacement curves were obtained from all runs. Typical load-time curves from the two mechanical tests are presented in Fig. 1. The others were similar. Note that displacement can be obtained by multiplying time with the rate of loading (0.5 mm/min). The curves from the DC test were much smoother and more linear from the point of initial contact between the specimen and the loading member up to the point of fracture. In contrast, those from the PO test had many load peaks and troughs throughout the loading history, with the load continuing to increase following the initial failure and a gradual reduction in load capacity after the peak load was reached.

### Bond strength of post-dentin disks

The bond strengths for the different groups are shown in Table 1. The results showed that the tensile bond strengths measured by the DC test (15.39 ± 2.00–20.01 ± 2.76 MPa) were higher than the shear bond strength determined by the PO test (5.14 ± 2.66–8.04 ± 3.93 MPa) for both of the materials and pretreatments used in this study (p = 0.000). On the other hand, the coefficients of variation of the tensile bond strength from DC (13.00–19.46%) were much lower than those of the shear bond strength given by the PO test (37.04–53.15%) for each of the cases.

**Table 1:** Bond strength (MPa) of post-dentin disks for each group.

	PBS				TC			
	Resin cement	Coefficients of Variation	RMGI	Coefficients of Variation	Resin cement	Coefficients of Variation	RMGI	Coefficients of Variation
DC	20.01 ± 2.76	13.79%	17.42 ± 3.39	19.46%	19.43 ± 2.48	12.76%	15.39 ± 2.00	13.00%
PO	8.04 ± 3.93	48.88%	6.75 ± 2.50	37.04%	5.87 ± 3.12	53.15%	5.14 ± 2.66	51.75%

The data shown are the means ± standard deviations. DC – diametral compression; PO – push-out test.

Resin cement produced significantly higher tensile bond strengths (19.43 ± 2.48 or 20.01 ± 2.76 MPa) than resin-modified glass ionomer (RMGI) (15.39 ± 2.00 or 17.42 ± 3.39 MPa) with (p = 0.000) or without (p = 0.011) thermal cycling (TC). After TC, the tensile bond strength of the RMGI group (15.39 ± 2.00 MPa) was significantly lower (p = 0.028) than that of the control group (17.42 ± 3.39 MPa), but there was no significant difference (p = 0.487) in the tensile bond strength of the resin cement specimens between the TC group and the PBS (phosphate-buffered saline) group (20.01 ± 2.76 and 19.43 ± 2.48 MPa). The shear bond strengths given by the PO test, on the other hand, presented no significant difference between any of the groups (8.04 ± 3.93, 6.75 ± 2.50, 5.87 ± 3.12 and 5.14 ± 2.66 MPa).

### Failure mode analysis

As shown in Table 2, resin cement and RMGI produced different failure modes in the mechanically tested specimens. With resin cement, adhesive failure between the cement and dentin was the most frequent failure mode in both the DC and PO tests. Without thermo-cycling, all the specimens failed at the cement-dentin interface. With thermo-cycling, 5% of the resin cement specimens showed mixed-mode failure, i.e., cohesive failure within the cement mixed with adhesive failure between the cement and dentin, in both the DC and PO test. In contrast, the RMGI disks showed predominantly mixed-mode failure in all four cases. The next major failure mode in the RMGI disks was adhesive failure between the cement and dentin, followed by adhesive failure between the post and cement, which was not seen in the resin cement specimens or RMGI specimens after thermal cycling.

**Table 2:** Number (percentage) of specimens failed with a particular fracture mode under the different experimental conditions.

Diametral compression test					Push-out test			
Failure Mode	PBS		**TC**		PBS		TC	
	Resin cement	RMGI	Resin cement	RMGI	Resin cement	RMGI	Resin cement	RMGI
CD	20(100)	9(45)	19(95)	5(25)	20(100)	5(25)	19(95)	7(35)
CP	0(0)	2(10)	0(0)	3(15)	0(0)	0(0)	0(0)	0(0)
Mixed	0(0)	9(45)	1(5)	12(60)	0(0)	15(75)	1(5)	13(65)

CD: adhesive failure between the cement and dentin; CP: adhesive failure between the cement and post; Mixed: cohesive failure within the cement mixed with adhesive failure between the cement and dentin/post.

### Interfacial leakage

**Figure 2:**
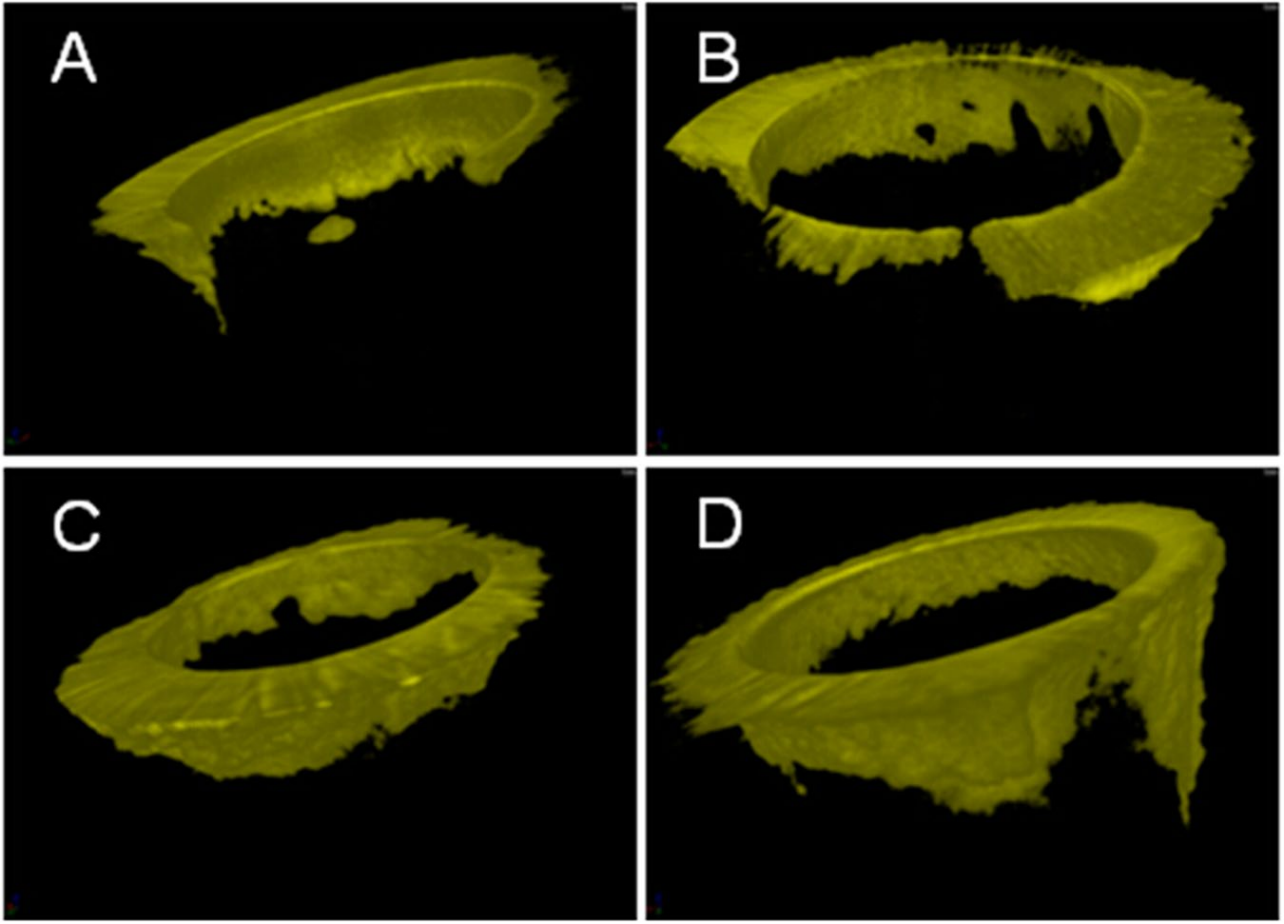
3D micro-CT images of AgNO_3_ that had infiltrated through the exposed dentin and along the post-dentin interface. (**A**) Resin cement with PBS. (**B**) Resin cement with TC. (**C**) RMGI with PBS. (**D**) RMGI with TC.

**Figure 3:**
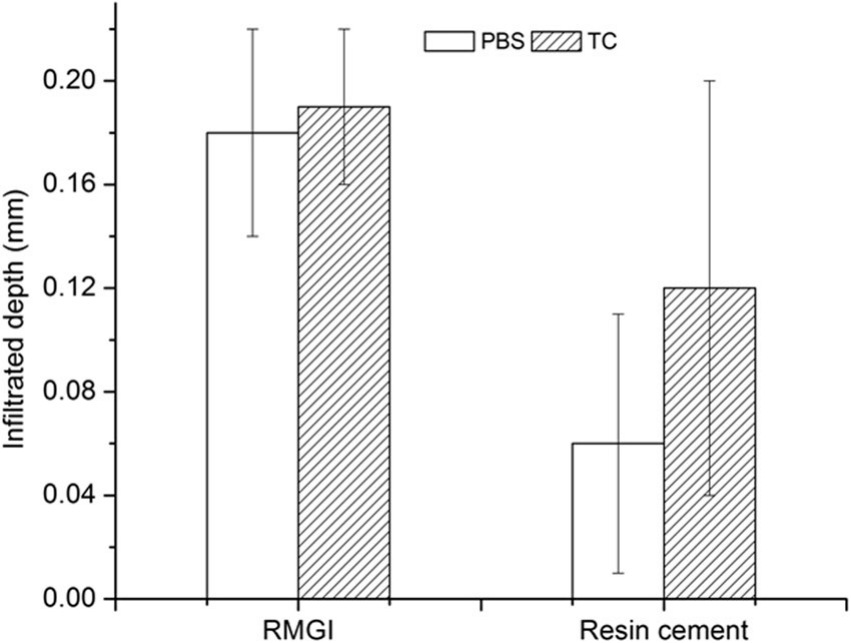
Depths of AgNO_3_ infiltration in post-dentin disks.

Figure 2 shows the micro-CT images of silver nitrate penetration in the post-dentin disks. The RMGI disks exhibited more interfacial leakage than the resin cement ones, with or without TC. In the RMGI disks, the infiltrated depth of silver nitrate with and without TC was 0.19 ± 0.03 mm and 0.18 ± 0.04 mm, respectively. In the resin cement disks, it was 0.12 ± 0.08 mm and 0.06 ± 0.05 mm, respectively (Fig. 3).

**Figure 4:**
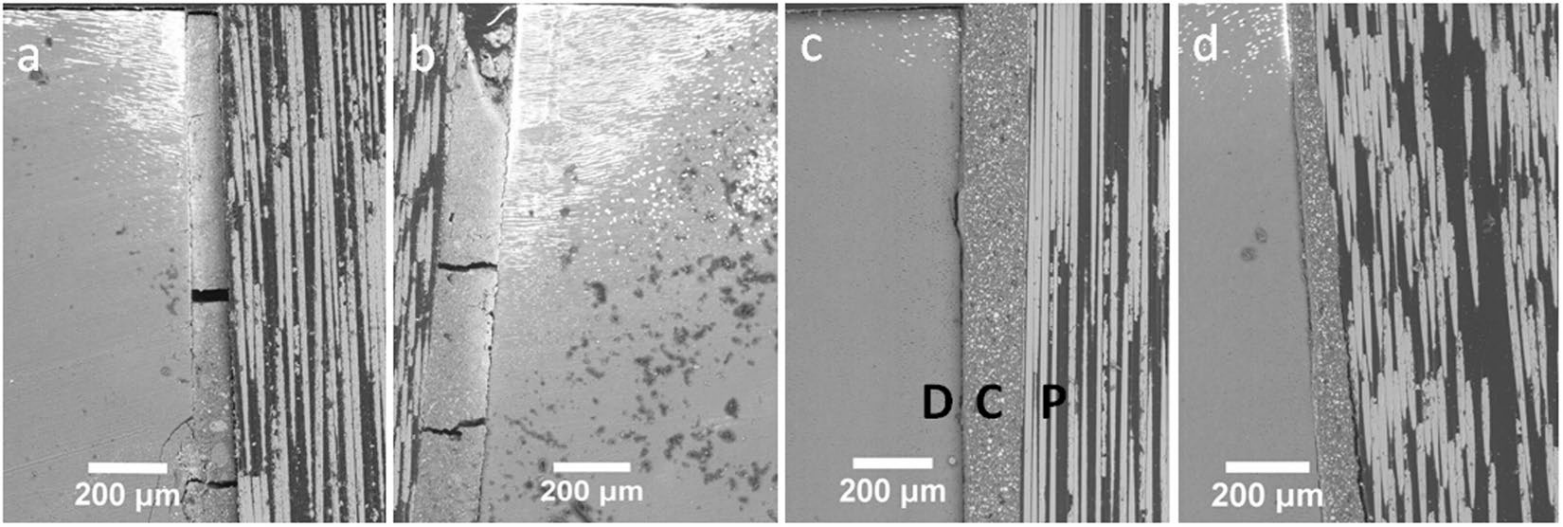
SEM images of post-dentin disks after silver nitrate penetration. (**a**) RMGI with PBS. (**b**) RMGI with TC. (**c**) Resin cement with PBS. (**d**) Resin cement with TC. P, post; C, cement; D, dentin.

SEM images of the post-dentin disks immersed in silver nitrate (Fig. 4) demonstrated that leakage started from the surface margin between the cement and dentin, propagated along the corresponding interface and then entered the dentinal tubules. Significant cracking can also be seen in the RMGI layer.

## Discussion

Based on the results from this study, the null hypothesis that there would be no significant difference between the post-dentin bond strengths measured by the two methods should be rejected.

Although human teeth were not used in this study, there are published papers^5,19–22^ which show that bovine teeth are good substitutes for human teeth, and the former are more readily available. While the measured bond strengths from using bovine teeth may be slightly different from those using human teeth, the main conclusion that diametral compression is a better test method than the push-out test is unlikely to depend on the tooth tissue used.

The DC test, as an indirect tensile test, induces a tensile stress in the direction transverse to the applied compressive load which splits the round specimen into two halves along the loading diameter and part of the post-dentin interface. By using digital image correlation and measuring acoustic emission, it has been shown that debonding at the post-dentin interface took place before fracture of the dentin in the disk specimen^17^. This is essential for the test to be valid for bond strength measurement. The load-displacement curves obtained from the DC test were much smoother and more linear up to the point of fracture when compared with those from the PO test (Fig. 1). Therefore, it was easier to pick out the final failure load from the DC test results, from which the post-dentin interfacial bond strength could be calculated using the load-stress relationship determined from the FEA^17^. In contrast, there were many load peaks and troughs on the curves from the PO test, making it hard to identify the failure load. Previous studies^14,15^ showed that friction between the post and dentin resulted in a stick-slip motion between them during loading beyond initial failure, which led to the many peaks and troughs on the load-displacement curves from the PO test. Another study further showed that the maximum peak load was controlled by the amount of friction between the post and dentin^23^, indicating that the effect of the material’s fracture properties was secondary. This may explain the small difference in the PO bond strength between the different groups. More importantly, FEA has shown that the stress along the interface is far from uniform, and the failure initiation stress is a combination of both shear and tension, the proportions of which being dependent on the material properties and dimensions of the different components of the specimen^18^. Therefore, the bond strength given by the PO test based on the simple formula cannot be regarded as a true material property.

**Figure 5:**
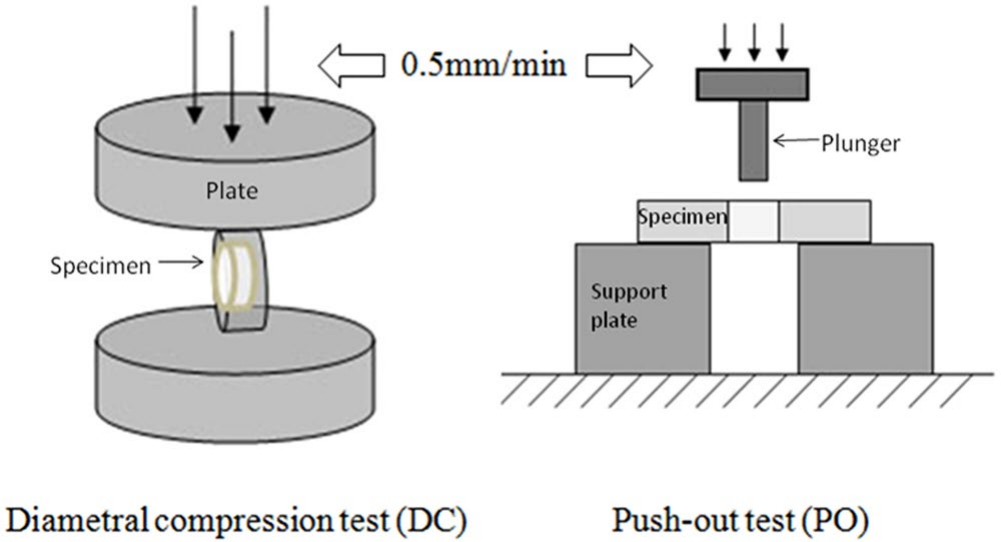
Schematic diagrams of the mechanical tests to determine post-dentin interfacial bond strength.

The DC test was much easier to perform compared to the PO test. As shown in Fig. 5, no alignment was necessary for the specimen in the DC test provided the two parallel horizontal plates are sufficiently rigid. For the PO test, on the other hand, the plunger, the post of the disk specimen, and the hole of the support plate all needed to be aligned concentrically (Fig. 5). A previous study^13^ also showed that the diameter of the plunger affected the push-out bond strength measured. Moreover, Fig. 1 shows that the DC test took less time to perform than the PO test (60 s *vs*. 300 s).

As presented in Table 1, the standard deviations of the bond strength values given by the PO test were much higher than those given by the DC test. The coefficients of variation of the bond strength given by the DC method were no more than 20%, which were in accordance with those from a previous study^17^. In contrast, the coefficients of variation of the bond strength from the PO test were around 50%, which also agreed with other studies^10–12^. Therefore, the PO technique could be less discriminatory in measuring the bond strength of different restorative systems. Stick-slip motions due to friction are highly nonlinear and history-dependent, which may explain the large variability in the PO test results.

The mean values of the tensile bond strength from the DC test were higher than the shear bond strength from the PO test. It may be true that the shear bond strength of these restorative systems is lower than their tensile bond strength. However, as mentioned above, the stress distribution along the post-dentin interface in the PO test specimen is far from uniform, with stress concentrations occurring near the free edges. The actual failure-causing stress, which is tensile, could therefore be much higher than the average shear stress given by the simple formula. This difference in stress distribution may explain why different measurement methods could lead to different values of the same bond strength^10^. If the maximum tensile stress was used instead to calculate the PO bond strength, values closer to those given by the DC test might be obtained.

**Figure 6:**
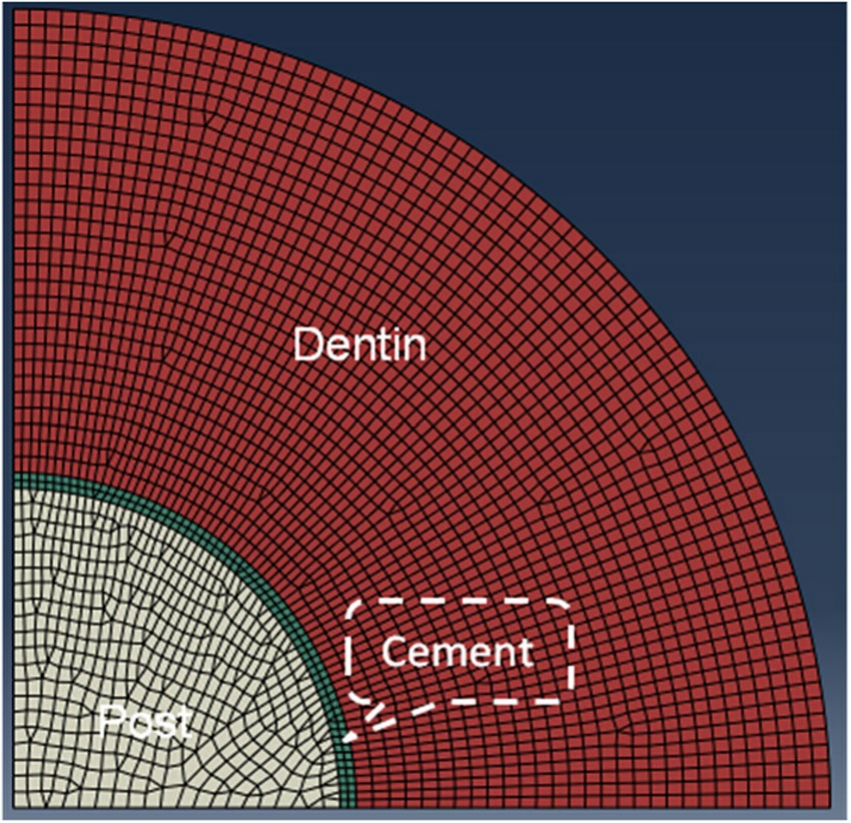
FEA model for the diametral compression (DC) test. Due to symmetry, only a quarter of the disk needs to be modeled.

A major disadvantage of the DC test is that FEA (Fig. 6) is required to calculate the tensile bond strength from the failure load. In contrast, a simple formula is used with the PO test to calculate the shear bond strength. However, as mentioned, the stress distribution in the PO test specimen is not that of simple shear. As a result, it will also be affected by the geometrical and material parameters^13^, and FEA is required to calculate the correct bond strength.

Aging could affect the post-dentin bond strength. In this study, TC was used to simulate the clinical challenge *in vitro*. There were no statistically significant differences in the shear bond strengths given by the PO test between the groups due to the large coefficients of variation of the results, even though reduction by TC was observed. In contrast, the tensile bond strength of the thermo-cycled RMGI group given by the DC test was significantly lower than that of the group without TC. Specimens prepared with resin cement appeared to show stronger resistance to thermal degradation. The better discriminatory power of the DC method was mainly due to the lower coefficients of variation in its measured bond strength. A previous study also showed that the bond strengths of various resin cements to root canal dentin using fiber-reinforced composite posts were significantly reduced by TC^24^.

The failure-mode analysis revealed that failure occurred predominantly at the interface between the cements and dentin. The same result was found in other similar studies^24–27^. The mixed failure mode was also common in the RMGI specimens, probably because RMGI is relatively brittle, as shown by the cracks in the RMGI layer (most likely due to drying of the specimens) in the SEM images (Fig. 4). Debonding between the post and dentin increases the risk of failure of endodontically treated teeth^3^, which could be accelerated by interfacial leakage. Micro-CT was used to assess interfacial leakage because it is non-destructive, quantitative and 3D in its analysis. The results from micro-CT and SEM revealed that interfacial leakage took place between the cements and dentin, and the volume of silver nitrate penetration in the RMGI disks were much higher than that in the resin cement disks. One reason for this difference could be that RMGI is a high-shrinkage cement, which resulted in premature interfacial debonding during setting. Therefore, the cement-dentin interface seemed to be the weakest part of the restorative system, where the majority of failures initiated.

It may be of interest to compare with the pull-out test, which is another popular test for bond strength testing^28–30^. It is considered that pull-out test is the best test according to distribution of stress^30^. However, a large number of fiber posts have to be used to prepare specimens in pull-out test because a few millimeters of the post out of the root canal should be leave for pulling action, which leads to the high cost of the experiment^28,29^. While for DC test and PO test, the roots filled with fiber posts were trimmed and sliced into disks, which decreased the use of the fiber posts in our study. On the other side, it is true that the ‘pull-out’ test may suffer less from post-failure interfacial friction because of the taper of the post. However, similar to the push-out test, failure of its specimens is likely to be caused by tensile, rather than shear, stress around the surface margin. It is also well known in experimental mechanics that tensile tests are more problematic due to errors associated with alignment and local stresses at the attachment points. The actual stress distribution and, hence, failure load will therefore depend on the geometry and mechanical properties of the materials that form the specimen. As shown in Fig. 5, the specimens used in our study are easier to perform than the specimens used in pull-out test in other studies^28,30^. Clinically, all teeth are subjected to compressive occlusal loads, mixed with some shear or bending, the degree of mixture being dependent on whether they are anterior or posterior. But they are never subjected to simple pull-out or tensile load, except during extraction. Of course, further studies are necessary to carry out to compare the pull-out test with DC test in the future.

## Methods

### Preparation of post-dentin disks

Forty bovine incisors, which were cleaned by removing the soft tissues and stored in 0.1% thymol solution at 4 ° C, were used to prepare the specimens. The crown portion above the cementum-enamel junction (CEJ) and a 3-mm long apical portion of the incisors were cut off with a low-speed diamond saw (Isomet, Buehler, Lake Bluff, IL, USA). The root canals were enlarged to a 2-mm parallel post space with a lower speed drill, and then the remaining roots were trimmed into cylinders with an outer diameter of 5 mm. The cylinders were then randomly divided into two groups and inserted with the cylindrical part of fiber posts (RelyX^TM^ Fiber Posts, 3M ESPE, St. Paul, MN, USA) that were luted with resin cement (RelyX^TM^ Unicem 2, 3M ESPE, St. Paul, MN, USA) or RMGI (RelyX^TM^ Luting Plus, 3M ESPE, St. Paul, MN, USA), following the manufacturer’s protocol. Finally, the restored cylinders were sliced into 2-mm thick disks, and then stored in de-ionized water at 4 °C no more than 24 h before testing. The bovine teeth were obtained commercially. All methods were carried out in accordance with the relevant guidelines and regulations.

### Thermo-cycling (TC)

Twenty post-dentin disks from each group were thermo-cycled using a two-bath thermo-cycler (Sabri Dental Enterprises, IL, USA) for 5000 cycles between 5 °C and 55 °C with a dwell time of 30 s in each bath and a transfer time of 10 s between them. After TC, the disks were collected for mechanical tests. Control groups of post-dentin disks of the same sample size were stored in PBS buffer at 37 °C for the same duration as that of TC prior to mechanical testing.

### Mechanical tests

The interfacial bond strengths of the post-dentin disks were determined using a universal test machine (858 Mini Bionix II, MTS, MN, USA).

DC was performed with two parallel horizontal steel plates, as shown in Fig. 5. The load was applied in a stroke-control mode at a loading speed of 0.5 mm/min. The fracture loads of the disks were determined from the load-displacement curves.

For the PO test (Fig. 5), each disk was positioned on a metal support plate with a central opening (ø = 3 mm) larger than the inner diameter of the disk. The post segment was loaded with a metallic cylindrical plunger (ø = 1.5 mm), also at a speed of 0.5 mm/min.

### Bond strength calculation

For the DC test, the relationship between the maximum post-dentin interfacial tensile stress and the force applied to the disk was established by using FEA, as shown in Fig. 6. 3D finite element models were created using the commercial software HyperMesh (version 11.0, Altair, Troy, MI, USA). The fiber post was considered transversely isotropic and a cement layer of 50 µm thick was also simulated in between dentin and fiber post. Young’s moduli for dentin, fiber post and cement were assumed to be 18.6, 9.5 and 7.7 GPa, respectively^17^. Poisson’s ratios for them were 0.31, 0.27 and 0.35, respectively^17^. The models were analyzed by the software ABAQUS (version 6.10, Rising Sun Mills, Providence, RI, USA). Due to symmetry, only a quarter of the disk needed to be modeled. The materials and dentin were considered homogeneous, linear elastic and isotropic. Appropriate boundary conditions (zero displacements in the normal direction) were prescribed along the vertical and horizontal diameters. A downward point load was applied at the top of the disk to simulate the external load. The post-dentin tensile bond strength was calculated using the failure load from the load-displacement curve.

For the PO test, the shear bond strength was calculated using the formula P/πdh, where P is the failure load, d is the inner diameter of the disk, and h is the height of the disk.

### Assessment of interfacial leakage between post and dentin

Micro-CT in combination with a radiopaque dye (silver nitrate, AgNO_3_) was used to assess leakage through the post-dentin margin^31,32^. Three freshly prepared disks from each group were painted with an acid-resistant nail varnish, leaving one side of the post and a perimeter of ~0.3-mm wide around it exposed. The painted disks were submerged into a 50% (w/w) solution of AgNO_3_. After 24 h of storage at room temperature, they were taken out for micro-CT scanning (XT H 225, Nikon Metrology Inc., Brighton, MI, USA). The scanning parameters were 90 kV, 90 μA, 708 ms of exposure, 720 projections and 4 frames per projection. After scanning, 3D reconstruction was performed using the software CT Pro 3D (Nikon metrology, Inc., Brighton, MI, USA). A cylindrical volume of interest (VOI) was generated around the posts to calculate the volume of silver nitrate that had penetrated into the post-dentin disks.

### Scanning electron microscopy

Scanning electron microscopy (SEM) was used to examine the fracture modes of the disks subjected to mechanical testing and the path of interfacial leakage of those immersed in AgNO_3_. It was conducted in a tabletop scanning electron microscope (TM-3000, Hitachi, Japan) at an accelerating voltage of 15 kV and in combo observation mode. The fractured disks were fixed on an aluminum stub with conductive carbon tapes. The painted post-dentin disks (n = 3 per group) treated in AgNO_3_ solution, as performed in above, were sectioned longitudinally and polished with 1200 grit sandpapers before SEM scanning to determine the infiltration depth.

### Statistical analysis

Assumptions of normal distribution were evaluated by Kolmogorov-Smirnov test. Two tailed *t* test was carried out by R (3.3.1, R Core Team, Vienna, Austria) to compare the bond strength between the groups (resin cement *vs*. RMGI, with or without thermal cycling). The level of statistical significance was set at 0.05.

### Data availability statement

Materials and data are available from the corresponding authors.
